# Antileishmanial activity and immunomodulatory effect of *seco*subamolide, a butanolide isolated from *Nectandra oppositifolia* (Lauraceae)

**DOI:** 10.1590/1678-9199-JVATITD-2019-0008

**Published:** 2019-08-19

**Authors:** Thais A. da Costa-Silva, Geanne A. Alves Conserva, Andrés J. Galisteo, Andre G. Tempone, João Henrique G. Lago

**Affiliations:** 1 Center of Natural Sciences and Humanities, Federal University of ABC (UFBAC), São Paulo, SP, Brazil.; 2 Institute of Tropical Medicine, University of São Paulo (USP), São Paulo, SP, Brazil.; 3 Centre for Parasitology and Mycology, Adolfo Lutz Institute (IAL), São Paulo, SP, Brazil.

**Keywords:** Leishmania (L.) infantum chagasi, Antileishmanial, Nectandra oppositifolia, *Seco*subamolide, Immunomodulatory

## Abstract

**Background:**

Visceral leishmaniasis is a complex neglected tropical disease caused by *Leishmania donovani* complex. Its current treatment reveals strong limitations, especially high toxicity. In this context, natural products are important sources of new drug alternatives for VL therapy. Therefore, the antileishmanial and immunomodulatory activity of compounds isolated from *Nectandra oppositifolia* (Lauraceae) was investigated herein*.*

**Methods:**

The *n*-hexane extract from twigs of *N. oppositifolia* were subjected to HPLC/HRESIMS and bioactivity-guided fractionation to afford compounds **1** and **2** which were evaluated *in vitro* against *Leishmania* (*L.*) *infantum chagasi* and NCTC cells.

**Results:**

The *n*-hexane extract displayed activity against *L.* (*L.*) *infantum chagasi* and afforded *iso*linderanolide E (**1**) and *seco*subamolide A (**2**), which were effective against *L.* (*L.*) *infantum chagasi* promastigotes, with IC_50_ values of 57.9 and 24.9 µM, respectively. Compound **2** was effective against amastigotes (IC_50_ = 10.5 µM) and displayed moderate mammalian cytotoxicity (CC_50_ = 42 µM). The immunomodulatory studies of compound **2** suggested an anti-inflammatory activity, with suppression of IL-6, IL-10, TNF with lack of nitric oxide.

**Conclusion:**

This study showed the antileishmanial activity of compounds **1** and **2** isolated from *N. oppositifolia*. Furthermore, compound **2** demonstrated an antileishmanial activity towards amastigotes associated to an immunomodulatory effect.

## Background

Visceral leishmaniasis (VL) is a complex neglected tropical disease caused by two species of *Leishmania* parasites: *Leishmania* (*L.*) *infantum chagasi* and *Leishmania* (*L.*) *donovani*. In South and Central Americas, Mediterranean Basin, Middle East and Central Asia, the VL agent is *L.* (*L.*) *infantum chagasi* while in Africa and Asia the agent is *L.* (*L.*) *donovani* [1, 2]. According to Drug for Neglected Disease initiative (DNDi) annually there are 20.000 to 30.000 deaths attributed visceral leishmaniasis [3]. It is considered a prioritized infectious disease by the World Health Organization (WHO) due to the worldwide prevalence [1].

*Leishmania* parasites have elaborated escape mechanisms of immune response [4, 5]. *L.* (*L.*) *infantum chagasi* invades cells of the mononuclear phagocytic system; reach mainly macrophages of spleen, liver and bone marrow and causes irregular fever, anemia, hepatosplenomegaly, pancytopenia, weight loss, and hypergammaglobulinemia [6]. Nowadays there are limited options of therapy for the various forms of leishmaniasis. The available treatment is based on the application of pentavalent antimonials (sodium stibogluconate and meglumine antimoniate), drugs that have been used for more than seven decades [2, 7]. This therapy shows a number of disadvantages for the patients, including high toxicity, adverse effects, requirement of hospitalization for the drug administration, and continuous increase of resistance that leads to decreasing effectiveness [2, 8, 9]. The single available drug for VL treatment by oral administration is the miltefosine, an anticancer drug. The antileishmanial effect was discovered in the 90’s and in 2003 it was licensed for the treatment of VL [10]. Various antileishmanial mechanisms have been attributed to miltefosine: effects in the parasite membrane, mitochondrial alterations, and immunomodulatory action with Th1 response [11]. In this context, the search of new compounds against *L.* (*L.*) *infantum chagasi* that could elicit the host immune response could be an interesting strategy to kill the parasite inside the macrophages [12, 13]. Natural products are an important source of new metabolites and play an important role in drug discovery and development process. Thus, these compounds could be considered a starting point for drug development due to their structural diversity and pharmacological potential [12-19].

In order to identify new antileishmanial molecules from Brazilian flora, the aim of this work was to investigate the *in vitro* antileishmanial activity of the *n*-hexane extract of the twigs of *Nectandra oppositifolia* (“canela-ferrugem” or “canela-amarela” in Portuguese). *Nectrandra* sp. is known for its anti-inflammatory, analgesic, and antiprotozoal [anti-*L.* (*L.*) *donovani* and anti-*Trypanosoma cruzi*] properties [19]. The genus *Nectandra* belongs to the Lauraceae family, which is composed of 2,500-3,000 species distributed in 49-50 genera. The distribution of this family occurs in tropical and subtropical regions of the world, predominantly in Southeast Asia and Brazil [20]. The studied extract was 100% active against promastigotes and amastigotes forms of *L. (L.) infantum chagasi* at the concentration of 200 µg/mL, then it was subjected to HPLC/HRESIMS analysis that indicated the predominance of two related metabolites: *iso*linderanolide E (**1**) and *seco*subamolide A (**2**). After purification over successive chromatographic steps, the effects of compounds **1** and **2** against *L.* (*L.*) *infantum chagasi* in macrophages and toxicity against mammalian cells were evaluated. Additionally, the immunomodulatory activity of active compound **2** was also assessed.

## Methods

### General experimental procedures

^1^H and ^13^C NMR spectra were registered at 500 and 125 MHz, respectively, on a Varian INOVA 500 spectrometer using CDCl_3_ as solvent and TMS as internal standards. Optical rotations were measured with a JASCO DIP-370 digital polarimeter. HPLC/HRESIMS analysis was performed on a Thermo Scientific Dionex UltiMate DAD 3000 detector and a Phenomenex Luna C-18 column (250 x 4.6 mm, 5 μm) coupled on a Bruker Daltonics MicroTOF QII spectrometer using an Apollo ion source set as follows: dry temperature at 180°C and voltage at 4.5 kV. The mass/charge ratios were detected in scan (*m/z* 100-1200 Da) and product ion scan (*m/z* 50-1200 Da) modes. Chromatographic separation procedures were performed using ACN:H_2_O 95:5 as eluent with a flow rate 1.0 mL/min and detection at 235 nm. For all extraction and chromatography procedures, were used analytical grade solvents.

### Plant material

*N. oppositifolia* twigs were collected in the Atlantic Forest area of Arthur Nogueira city, São Paulo State (coordinates 22º30'57,65''S, 47º10'50,11'' W), Brazil, in April 2016. The plant material was identified by MSc. Guilherme M. Antar and a voucher specimen (SPF 225339) has been deposited in the Herbarium of Institute of Biosciences, University of São Paulo, SP, Brazil.

### Extraction

*N. oppositifolia* twigs were dried, powdered (310 g) and exhaustively extracted using *n*-hexane at room temperature. After evaporation of the solvent at reduced pressure, 3.4 g of *n*-hexane extract were obtained.

### HPLC/HRESIMS analysis

Part of crude *n*-hexane extract from twigs of *N. oppositifolia* (5 mg) was dissolved in 1 mL of MeOH and filtered on a C_18_ Sep-Pak. Sample containing 1 μL of crude extract was analyzed by HPLC/HRESIMS.

### Bioactivity-guided fractionation

Part of the crude *n*-hexane extract (3.0 g) from twigs of *N. oppositifolia* was chromatographed over a silica gel column, eluted with increasing amounts of EtOAc in *n*-hexane. This procedure yielded 60 fractions (100 mL each) that were combined into five groups (A-E) on the basis of similarities on TLC profiles. As the activity against promastigote forms of *L. (L.) infantum chagasi* was detected in group C (277 mg), part of this bioactive group (245 mg) was chromatographed over a silica gel column, eluted with a mixture of CHCl_3_:Me_2_CO 96:4 (v/v). This procedure resulted in eight groups (C1-C8) being activity against promastigote forms of *L. (L.) infantum chagasi* detected in groups C6 (54 mg) and C7 (32 mg). Part of group C6 (30 mg) was purified by RP-HPLC (eluent ACN:H_2_O 95:5) to afford pure compound **1** (6.0 mg). Part of bioactive group C7 (25 mg) was purified by silica gel prep. TLC (CHCl_3_:Me_2_CO 98:2) to afford pure compound **2** (3.4 mg). 

*Isolinderanolide E* (**1**). White amorphous solid. [α]_D_^25^ + 13.8 (*c* 0.20, CHCl_3_); ^1^H NMR (CDCl_3_, 500 MHz) δ 7.09 (1H, td, *J* = 7.9 and 2.2 Hz, H-6), 5.26 (1H, br s, H-3), 4.96 (1H, dd, *J* = 2.8 and 1.7 Hz, H-5a), 4.73 (1H, dd, J = 2.8 and 1.4 Hz, H-5b), 2.47 (2H, m, H-7), 1.53 (2H, m, H-8), 1.26 (34H, s, H-9 to H-20), 0.88 (3H, t, *J* = 6.9 Hz, H-21); ^13^C NMR (CDCl_3_, 125 MHz) δ 166.5 (C-1), 157.6 (C-4), 150.2 (C-6), 127.3 (C-2), 91.4 (C-5), 66.5 (C-3), 31.9 (C-19), 29.7 - 29.4 (C-9 to C-19), 29.6 (C-7), 28.3 (C-8), 22.7 (C-20), 14.1 (C-21).

Seco*subamolide A* (**2**). White amorphous solid. [α]_D_^25^ + 18.1 (*c* 0.12, CHCl_3_); ^1^H NMR (CDCl_3_, 500 MHz) δ 7.08 (1H, t, *J* = 7.6 Hz, H-6), 4.90 (1H, br s, H-3), 3.73 (3H, s, 1-OCH_3_), 2.35 (2H, t, *J* = 7.6 Hz, H-7), 2.15 (3H, s, H-5), 1.53 (2H, t, *J* = 7.6 Hz, H-8), 1.31 - 1.25 (34H, s, H-8 to H-20), 0.88 (3H, t, *J* = 7.0 Hz, H-21); ^13^C NMR (CDCl_3_, 125 MHz) δ 206.3 (C-4), 166.6 (C-1), 149.1 (C-6), 129.2 (C-2), 73.4 (C-3), 52.0 (1-OCH_3_), 31.9 (C-19), 29.7 - 29.4 (C-9 to C-18), 28.9 (C-7), 28.7 (C-8), 24.8 (C-5), 22.7 (C-20), 14.1 (C-21).

### Experimental animals

The experimental animals used in this study, golden hamsters (*Mesocricetus auratus*) and BALB/c mice, were supplied by the *Instituto Adolfo Lutz* of São Paulo State, Brazil. The animals received food and water *ad libitum* and maintained in sterile boxes. Golden hamsters were inoculated every month with amastigotes purified from spleen derived of a previously infected hamster, for the maintenance of the *L.* (*L.*) *infantum chagasi* strain. BALB/c mice were used as a source of peritoneal macrophages. Animal procedures were conducted with the approval of the Ethics Committee of Instituto Adolfo Lutz (project CEUA-IAL/Pasteur 05/2011) in accordance with the National Institutes of Health “Guide for the Care and Use of Laboratory Animals” (NIH Publications No. 8023).

### *L. (L.) infantum chagasi* promastigotes, peritoneal macrophages, and NCTC cell culture 

*L.* (*L.*) *infantum chagasi* parasites (MHOM/BR/1972/LD) were maintained through successive passages in golden hamsters up to 60-70 days after infection. The amount of parasites in the spleen was determined 60-70 days post infection [21]. Promastigotes forms were kept in cell culture flasks in M-199 medium supplemented with 10% fetal calf serum (FCS) and 0.25 % hemin at 24 ºC BOD incubator. Macrophages were obtained from the peritoneal cavity of BALB/c mice by washing with RPMI-1640 medium supplemented with 10% FCS and kept at 37ºC in a 5% CO_2_-humidified incubator [22]. Murine fibroblasts NCTC cells (clone L929 ATCC) were kept in cell culture flasks in M-199 medium supplemented with 10% FBS and 20 µg/mL gentamicin at 37ºC in a 5% CO_2_-humidified incubator.

### Evaluation of 50% inhibitory concentration (IC_50_) against *L.* (*L.*) *infantum chagasi* and 50% cytotoxicity concentration (CC_50_) against NCTC cells

To determine the IC_50_ concentration against promastigotes forms of *L.* (*L.*) *infantum chagasi* the crude *n*-hexane extract from *N. oppositifolia*, fractions and compounds **1** and **2** were dissolved in DMSO and diluted in M-199 medium in 96-well microplates. Extract/fractions were tested at highest concentration of 200 µg/mL while purified compounds were serially tested at the concentrations of 150 to 1.71 µM. The promastigotes (late growth phase) were counted in a Neubauer chamber and seeded at 1 x 10^6^/well and incubated with the compounds in the different concentrations for 48 h at 24ºC in a BOD incubator. The viability of the parasites was evaluated using the MTT reagent [23]. Miltefosine was used as standard drug. An internal control group was used with 0.5% DMSO (maximal concentration).

To determine the CC_50_ concentration, compounds **1** and **2** were dissolved and diluted as described above. Thus, compounds were serially tested at the concentrations of 200 to 1.56 µM. NCTC cells were scrapped from the cell culture flasks counted in a Neubauer chamber and seeded at 6 x 10^4^ cells/well and incubated with compounds in the different concentrations for 48 h at 37ºC in a 5% CO_2_-humidified incubator. The viability of the cells was evaluated using the MTT reagent [23].

To determine the IC_50_ concentration against intracellular forms of *L.* (*L.*) *infantum chagasi* (amastigotes), macrophages collected from the peritoneal cavity of BALB/c mice were counted in a Neubauer chamber, seeded at 1 x 10^5^/well in a 16-well slide and kept in a 5% CO_2_-humidified incubator overnight. Posteriorly, amastigotes were collect from a previously infected hamster as described, seeded at a ratio 1:10 (macrophages:amastigotes) and maintained at 37(C in a 5% CO_2_-humidified incubator for 24 h. Subsequently, crude extract and fractions were incubated with the infected macrophages to the highest concentration of 200 µg/mL while pure compounds **1** and **2** were tested at range 100 to 1.56 µM with infected macrophages for 96 h. Miltefosine was used as a standard drug. Last step of the assay, the macrophages were fixed with MeOH, stained with Giemsa (Merck KGaA, Germany), and analyzed on a light microscope. The parasite burden was determined by the number of infected macrophages out of 200 cells.

### Quantification of cytokine production by macrophages

Peritoneal macrophages from a BALB/c mice were counted in a Neubauer chamber and seeded in 24-well plates at 1 x 10^5^ cells/well in RPMI medium supplemented with 10% FBS and incubated for 24 h at 37ºC in a 5% CO_2_-humidified incubator. Next, the macrophages were washed with PBS and infected (overnight) with *L.* (*L.*) *infantum chagasi* amastigotes (ratio 10:1). Subsequently, cells were washed with PBS and treated with compound **2** (at IC_50_ value) for 48 h. Lipopolysaccharides (LPS) from *Escherichia coli* (Sigma-Aldrich, USA) was used as a positive control of production of cytokines. Non-infected macrophages were also treated with compound **2** in order to compare them with the treatment for infected cells. The cytokines levels were analyzed in culture supernatants after 48 h post treatment. The concentration of interleukin-6 (IL-6), interleukin-10 (IL-10), monocyte chemoattractant protein-1 (MCP-1) and tumor necrosis factor (TNF) was determined using an inflammatory CBA kit assay (BD Mouse Inflammation Kit, USA) in accordance with manufacturer protocols by flow cytometry (BD-LSR FORTESSA).

### Quantification of nitric oxide (NO)

To determine the NO concentration, peritoneal macrophages collected as described above were treated with compound **2** for 48 h at IC_50_ concentrations. The quantification of NO was determined by the Griess assay [24] in the supernatants of treated cells. LPS from *Escherichia coli* (Sigma-Aldrich, USA) was used as a positive control of production of NO. Obtained data were calculated from a standard curve prepared with NaNO_2_ in concentrations ranging from 1 to 400 μM.

### Statistical Analysis

The results were reported as the mean and standard deviation of duplicate samples from two or three independent assays. IC_50_ and CC_50_ values were calculated using sigmoid dose-response curves in Graph Pad Prism 5.0 software (Graph Pad Software, USA), ANOVA for significance (p < 0.05).

## Results and Discussion

**Figure 1.**
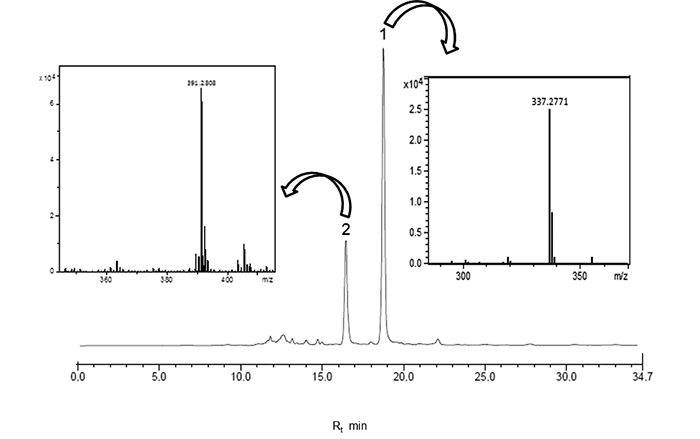
HPLC/HRESIMS analysis of crude *n*-hexane extract from twigs of *N. oppositifolia*.

**Figure 2.**
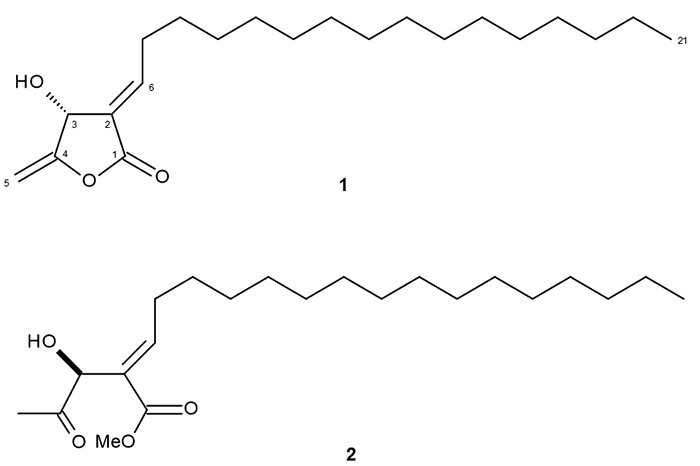
Structures of compounds **1** and **2** isolated from twigs of *N. oppositifolia.*

The parasitic activity of the *n*-hexane extract of the twigs of *N. oppositifolia* was determined against *L.* (*L.*) *infantum chagasi* (promastigote and amastigote forms), causing 100% of parasite death at 200 μg/mL. Aiming at the identification of bioactive compounds, the crude extract was analyzed by HPLC/HRESIMS and two main peaks were detected (Figure 1). Mass spectra analysis suggested the occurrence of two related butanolides due to the *quasi*-molecular ion peaks at *m/z* 337.2771 [M + H]^+^ and 391.2808 [M + Na]^+^, corresponding to molecular formulas C_21_H_36_O_3_ (compound **1**) and C_22_H_40_O_4_ (compound **2**), respectively. After successive chromatographic steps, compounds **1** and **2** were isolated in 99% of purity as indicated by HPLC. Structures of *iso*linderanolide E and *seco*subamolide A (Figure 2) were confirmed by analysis of ^1^H and ^13^C NMR spectra and comparison with data reported in the literature [25, 26].

*In vitro* antileishmanial activity of the isolated butenolides against the promastigotes and amastigotes forms of the *L.* (*L.*) *infantum chagasi* and mammalian cytotoxicity (NCTC cells) were evaluated. Butanolides **1** and **2** were effective against *L.* (*L.*) *infantum chagasi* promastigotes, with IC_50_ values of 57.9 ± 15.4 and 24.9 ± 2.8 µM. Considering the effect against amastigotes, compound **2** was effective with an IC_50_ value of 10.5 ± 2.3 µM. Compound **2** was more toxic than the standard drug (miltefosine) and it was specifically more effective against intracellular amastigote forms of the parasite (Table 1). As previously reported [27], lactones with an aliphatic side chain displayed activity and reduced cytotoxicity against amastigote forms of *Leishmania* sp. Another important structural aspect of this activity is the presence of the α, β-unsaturated lactone moiety since saturated derivatives showed reduced activity [28]. Our results indicated that despite compound **1** exhibit these structural characteristics described above, it was active only against the extracellular promastigotes. Otherwise, it is biosynthetically related to compound **2**, which showed an opening lactone unity, but maintaining the α,β?unsaturated system. Compound **2** showed activity against the extracellular (promastigote) and intracellular (amastigote) forms of *L. (L.) infantum chagasi.* Therefore, this is an important structural aspect to be considered for the future synthesis of new related bioactive derivatives.

**Table 1.** Antileishmanial activity (promastigote and amastigote forms) and mammalian cytotoxicity (NCTC cells) of compounds **1** and **2**, isolated from *N. oppositifolia*

Compounds	*L. (L.) infantum chagasi*		NCTC	SI^c^
	IC_50_^a^ µM (95% CI)		CC_50_^b^ µM (95% CI)	Amastigote
	*Amastigote*	*Promastigote*		
1	NA	57.9 ± 15.4	> 200	_
2	10.5 ± 2.3	24.9 ± 2.8	42.3 ± 14.8	4.0
Miltefosine	17.8 ± 1.4	16.7 ± 3.5	> 200	> 11.2

a: 50% inhibitory concentration, ^b^: 50% cytotoxic concentration, ^c^: Selectivity Index in intracellular amastigote forms; 95% CI: 95% confidence interval; NA: non active.

**Figure 3.**
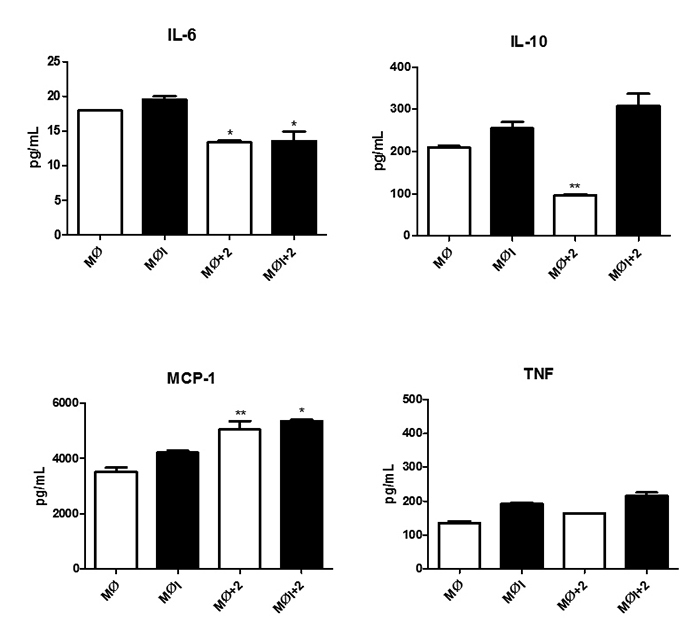
Effects of treatment of *L.* (*L.*) *infantum chagasi*-infected macrophages with *seco*subamolide A (compound **2**) on the production of proinflammatory cytokines (TNF, MCP-1, IL-6, and IL-10). LPS was used as positive control, and macrophages without treatment were used as negative control. The results are expressed in pg/mL, and the mediators were measured by flow cytometry (BD-LSR FORTESSA) in the culture supernatants with the CBA kit assay (BD Mouse Inflammation Kit, USA). MØ: macrophages, MØI: *Leishmania*-infected macrophages. (**IL-6**) LPS production: MØ 8737 pg/mL, MØI 9060 pg/mL. (**IL-10**) LPS production: MØ 859 pg/mL, MØI 955 pg/mL. (**MCP-1**) LPS production: MØ 4769 pg/mL, MØI 5301 pg/mL. (**TNF**) LPS production: MØ 4973 pg/mL, MØI 5260 pg/mL.

Considering the activity of the compound **2** against the amastigotes inside macrophages, the immunomodulatory effect in *Leishmania*-infected cells was also evaluated. The mechanism of cellular death of intracellular amastigotes of *Leishmania* could be an event associated to the activation of microbicide mechanisms of the macrophages, particularly the increasing of production of NO levels [29]. Different natural products such as sesquiterpene lactones, alkaloids and neolignans demonstrated this effect [13, 30, 31]. Compound **2** showed no induction of NO production in macrophages (data not shown). Furthermore, the analysis of the cytokine profile of infected and non-infected macrophages after treatment with compound **2** demonstrated an anti-inflammatory profile (Figure 3). This compound negatively modulated the production of one of the cytokine that is related to disease progression, IL-6, in infected and non-infected macrophages [12,32, 33]. Moreover, this compound did not increment IL-10 in statistically significant levels in infected macrophages, which favors treatment, since IL-10 is also related to VL progression [33-35,]. In addition, compound **2** increased the production of MCP-1 (CCL-2). CCL-2 is an important chemokine associated with the reduction of the parasite load and granuloma formation in the liver in experimental model of VL [36-39], with no interference in TNF levels, which could be related to the absence of NO. Higher levels of TNF are involved in macrophage activation and upregulation of iNOS expression, leading to the upregulation of NO levels [36]. Then, a possible antileishmanial effect of *seco*subamolide A may involve the suppression of IL-6 and increase of MCP-1.

## Conclusion

This work showed, for the first time in the literature, the anti-*Leishmania* (*L.*) *infantum chagasi* activity of related butanolide *iso*linderanolide E (**1**) and *seco*subamolide A (**2**) isolated from *N. oppositifolia*. Furthermore, compound **2** demonstrated an anti-*L. (L.) infantum chagasi* activity towards the most relevant parasite form, associated to an immunomodulatory effect in the host cells. The moderate selectivity found for this compound could be further improved in drug design studies. 

### Abbreviations

CC_50_: 50% cytotoxicity concentration; DNDi: Drug for Neglected Disease initiative; FCS: fetal calf serum; IC_50_: 50% inhibitory concentration; IL-10: interleukin-10; IL-6: interleukin-6; LPS: lipopolysaccharides; MCP-1: monocyte chemoattractant protein-1; NO: nitric oxide; TNF: tumor necrosis factor; VL: visceral leishmaniasis.
